# Poor food and nutrient intake among Indigenous and non-Indigenous rural Australian children

**DOI:** 10.1186/1471-2431-12-12

**Published:** 2012-02-04

**Authors:** Josephine D Gwynn, Victoria M Flood, Catherine A D'Este, John R Attia, Nicole Turner, Janine Cochrane, Jimmy Chun-Yu Louie, John H Wiggers

**Affiliations:** 1 Faculty of Health, University of Newcastle, Callaghan 2308 NSW Australia; 2 Cluster for Public Health Nutrition, Boden Institute of Obesity, Nutrition, Exercise and Eating Disorders, University of Sydney, Sydney, NSW 2006, Australia; 3 School of Health Sciences, Faculty of Health and Behavioural Sciences, The University of Wollongong NSW 2522, Australia; 4 Durri Aboriginal Medical Service Corporation, Kempsey, NSW 2440, Australia; 5 Biripi Aboriginal Medical Service, Taree, NSW 2430, Australia

## Abstract

**Background:**

The purpose of this study was to describe the food and nutrient intake of a population of rural Australian children particularly Indigenous children. Participants were aged 10 to 12 years, and living in areas of relative socio-economic disadvantage on the north coast of New South Wales.

**Methods:**

In this descriptive cross-sectional study 215 children with a mean age of 11.30 (SD 0.04) years (including 82 Indigenous children and 93 boys) completed three 24-hour food recalls (including 1 weekend day), over an average of two weeks in the Australian summer of late 2005.

**Results:**

A high proportion of children consumed less than the Australian Nutrient Reference Values for fibre (74-84% less than Adequate Intake (AI)), calcium (54-86% less than Estimated Average Requirement (EAR)), folate and magnesium (36% and 28% respectively less than EAR among girls), and the majority of children exceeded the upper limit for sodium (68-76% greater than Upper Limit (UL)). Energy-dense nutrient-poor (EDNP) food consumption contributed between 45% and 49% to energy. Hot chips, sugary drinks, high-fat processed meats, salty snacks and white bread were the highest contributors to key nutrients and sugary drinks were the greatest *per capita *contributor to daily food intake for all. *Per capita *intake differences were apparent by Indigenous status. Consumption of fruit and vegetables was low for all children. Indigenous boys had a higher intake of energy, macronutrients and sodium than non-Indigenous boys.

**Conclusions:**

The nutrient intake and excessive EDNP food consumption levels of Australian rural children from disadvantaged areas are cause for concern regarding their future health and wellbeing, particularly for Indigenous boys. Targeted intervention strategies should address the high consumption of these foods.

## Background

Indigenous peoples internationally suffer greater early mortality rates and poorer health status when compared with non-Indigenous peoples [1]. In Australia this gap is greater than for any other similar country, and particularly so for chronic diseases [1-3]. Rates of diabetes for Indigenous peoples are at least 3 times that of non-Indigenous Australians [3], and are especially high for Indigenous youth (6 times higher than for non-Indigenous youth) [4,5]. Poor nutritional status both *in utero *and during childhood is recognised as a key risk factor for the development of type 2 diabetes [2], and improving the diet of children is an acknowledged strategy for reducing the risk of chronic diseases during childhood and in adulthood [6].

Similar to that of Indigenous populations internationally [7], dispossession of Australia's Indigenous peoples has contributed to endemic disadvantage [3,8] and poor nutrition [3,9]. This is associated with the change of dietary patterns that occurred with European invasion, from consumption of traditional nutrient dense, low energy foods [2] to a dependence on poorer quality food handouts of staples such as white flour, sugar and rice [2]. Since then food intake for Indigenous peoples has been further compounded by many factors [8] including inadequate food access and availability [3,10], food insecurity [11] and financial stress [12], the last identified as a substantial barrier to a healthy diet [13,14].

It is acknowledged that good quality health data from Indigenous populations internationally are limited [8] and this is also true for Australia's Indigenous peoples [7,15], and for children and youth in particular [15,16]. Whilst the poorer nutritional status of Indigenous peoples relative to non-Indigenous peoples has been documented [5,7], internationally and nationally there are few studies comprehensively examining their food and nutrient intake [7,17], and even fewer involving children [2,7]. This represents a critical gap in the knowledge base required to develop effective health management strategies for this at-risk population [18].

The purpose of this study is to describe the food and nutrient intake of a population of Australian Indigenous and non-Indigenous rural children aged 10 to 12 years old, and who live in regions of relative social disadvantage, by examining 1) their mean daily intake of micro- and macro-nutrients and the percent contribution of macronutrients to energy; 2) the proportion of children with mean daily intakes of selected nutrients less than the estimated average requirement (EAR) or greater than the Upper Level (UL) of intake, as appropriate, and 3) the main food groups and sub-groups contributing to energy, fat, saturated fat, sugar, sodium and fibre.

Indigenous communities who participated in this research prefer the term 'Aboriginal and Torres Strait Islander'. This term is used from here on.

## Methods

### Setting

This descriptive cross-sectional study was undertaken in 3 regional areas on the north coast of the Australian state of New South Wales (NSW) in the summer of late 2005 and early 2006.

### Participants

In total, 11 Department of Education and Training ('government') primary schools were selected to participate in this research. These schools were chosen as they had the highest enrolments of Aboriginal and Torres Strait Islander children in their areas. All schools were located in local government areas defined as areas of relative socio-economic disadvantage [19,20].

All children in years 5 and 6 at the selected schools in 2 of the areas were invited to participate, and in the third area only Aboriginal and Torres Strait Islander children were invited to ensure an adequate sample from this population. Aboriginal Health Workers (AHWs) (Aboriginal and Torres Strait Islander people who are employed to work with Indigenous communities regarding all aspects of health care) co-ordinated the information and consent process within their communities.

### Measures and Data Collection Procedures

#### Height and Weight

Research assistants recorded demographic information for each child including gender, date of birth and Indigenous status. Height and weight were measured and body mass index (BMI) calculated (kg/m^2^) [21]. This information was used in the assessment of plausibility of the nutrition data.

#### 24-hour Food Recall

Each participant was invited to complete three 24-hour food recalls (including 2 weekdays and 1 weekend day) over an average of a 2-week period. The multiple-pass 24-hour recall method [22] was used by research assistants, including AHWs, who had been trained in the technique by the study research nutritionist. The 24-hour recalls were conducted as face to face interviews with each participant during school time and involved the use of prompts such as food models and food packaging. Information pertaining to the brand, type and weight of each item (for example Dairy Farmers skim milk 250 g) were obtained from respondents.

#### Physical Activity

Physical activity data were self-reported by the respondents using the Many Rivers Physical Activity Recall Questionnaire (MRPARQ), described elsewhere [23]. This information was used in the assessment of plausibility of the nutrition data.

#### Aboriginal and Torres Strait Islander children

This study is part of an Aboriginal community-initiated program of research. An Aboriginal and Torres Strait Islander community-controlled governance structure guided the study and AHWs had a high-profile role in the design and implementation of the study. AHWs who lived in the participating communities were employed to: facilitate recruitment of Aboriginal and Torres Strait Islander children, and support their completion of all study procedures; advise on data collection procedures; liaise with schools; take height and weight measurements; administer the 24-hour recalls for all Aboriginal and Torres Strait Islander children; and provide support to families and community as needed.

Ethics approval for the study was provided by the NSW Hunter Area Health Service, the NSW Mid North Coast Area Health Service, the University of Newcastle, the NSW Department of Education and Training and the Aboriginal Health and Medical Research Council of NSW. Parental informed consent and child assent were requirements for participation.

### Statistical Methods

Each participant's 24-hour recalls, gender, height and weight data were entered into the nutrient analysis package Foodworks Professional Version 4, using AusNut 1999 as the nutrient database [24]. All data were exported to a Microsoft Access database for cleaning. Statistical analyses were conducted with Statistical Packages for Social Science (SPSS) version 19.0 for height and weight data, and with SAS 9.1 and STATA Version 10 for all other data. Further information was added to the database including: Indigenous status; reported physical activity level (PAL) determined by information obtained from the physical activity survey [23]; and data obtained from food product labels and manufacturer information to complete the assessment of sodium and folate reflecting more recent changes to the food supply. This information was specific to brand names and also reflected the nutrient composition of fortified food items.

#### Food Groups

Foods were categorised into food categories, and items were based on questions from a separate short food frequency questionnaire described elsewhere [25]. The categories (most are listed in Additional Tables S3 and S4) were similar to 2007 Australian National Children's Nutrition and Physical Activity Survey categories [26] where possible, or represented common dietary eating habits, such as take-away food consumption.

#### Establishing Plausibility of Reported Nutrient Intake on Recall Records

The plausibility of participants reported nutrient intake was assessed by using the Goldberg cut-offs [27] for energy intake for their specific reported PAL as described by Black [28]. For participants without weight and/or height data (n = 6), their reported energy intake was compared with the range of reported energy intake of the participants in the corresponding PAL. For participants without physical activity data (n = 18), a light-activity PAL of 1.6 was assigned and the method described above using Goldbergs cut-offs for energy intake was repeated.

#### Statistical Analysis

##### Body Mass Index

An analysis of variance was conducted in SPSS to explore differences in mean BMI by gender and Indigenous status (ie whether Aboriginal and Torres Strait Islander or not).

##### Mean daily intake of micro- and macro-nutrients

The mean daily energy intake for each participant was calculated as an average of the 3 days of recall. T-tests adjusted for clustering of children within schools (for normally distributed data) and Kruskall-Wallis tests (for non-normally distributed data) were conducted to compare the mean daily nutrient intake and mean percentage contribution of all macro-nutrients to energy by Indigenous status within gender. Linear regression analyses, adjusted for clustering within schools, were conducted with the normally distributed data to examine the difference in each mean daily nutrient intake by Indigenous status adjusted for gender and age. Non-normally distributed data (vitamin A, beta-carotene, folate, vitamin C, calcium and zinc) were assessed using the Friedman's test. For non-parametric analysis, there is no method of adjustment for clustering of children within schools. Therefore, we obtained the design effect in order to estimate the likely impact of clustering on interpretation of the results.

##### Proportion of children not meeting Australian guidelines for mean daily intake of selected nutrients

The proportions of participants with mean daily intake of nutrients less than the EAR, less than the adequate intake (AI) for fibre and potassium or greater than the UL for sodium were calculated [29]. Chi-square tests were undertaken to compare these proportions by Indigenous status and by gender. Mantel-Haenszel tests were undertaken to examine proportions by Indigenous status adjusted for gender. The nutrients examined were those judged by the researchers to be of key importance to children's dietary intake, and reflect the nutrients commonly included in front-of-pack labelling of food products.

##### Highest ranked food categories and food items contributing to energy and selected nutrients

The total weight of each food category (for example 'breads') and item (for example 'white bread'), and their total constituent micro- and macro-nutrients were calculated by Indigenous status. The 'take-away food' category does not include the items of sugary drinks, hot chips, or chicken as these have their own separate categories. The take-away foods included mostly pizza and burgers, as well as spring rolls/dim sims and quiche. Mixed food items were each allocated a proportion, which was then included in the relevant recall food group calculations for weight and frequency. Each food group category was then ranked according to its percent contribution to total energy, fat, saturated fat, sugar, sodium and fibre. Linear regression, adjusted for clustering of children within schools, was conducted to compare the mean percent contribution of energy dense nutrient poor (EDNP) foods to energy by Indigenous status and gender. Daily *per capita *weight and weight per eating occasion of each food category were calculated. Food items were ranked according to their contribution by weight to the food categories. The *per capita *and per eating occasion weight of all food categories (and the weight of the highest ranked food item within) contributing to the above nutrients were then reported by Indigenous status. The percent contribution of EDNP food categories to energy were reported by Indigenous status within gender.

#### Sample Size

A 5% significance level was assumed, as was 80% power, a design effect of 1.5 for clustering of children within schools, and that approximately half of participants would be Aboriginal and Torres Strait Islander. With 250 children the study would be able to detect differences in nutrient intake between groups (by gender and Indigenous status) of approximately 0.5 of a standard deviation, and between Aboriginal and Torres Strait Islander and non-Indigenous children within each gender of 0.8 of a standard deviation.

## Results

**Figure 1:**
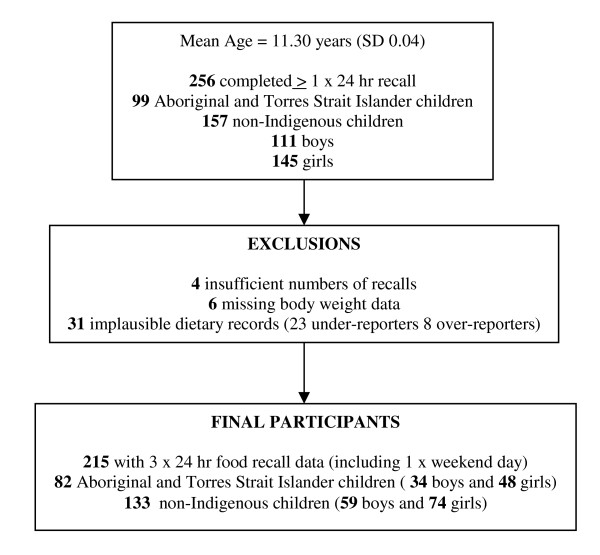
**Study Population**.

All 11 schools approached agreed to participate, yielding a total of 219 Aboriginal and Torres Strait Islander and 562 non-Indigenous children. The final study population comprised 259 children, a response rate of 47% (n = 102) of Aboriginal and Torres Strait Islander children and 28% (n = 157) of non-Indigenous children. Of these, 256 children completed at least one 24-hour recall, and 215 children completed three 24-hour recalls each (including one weekend day) and were included in the analysis (Refer to Figure 1).

### Body Mass Index

No statistically significant differences in BMI were found by Indigenous status for all children or by Indigenous status within gender (Table 1).

**Table 1:** Mean (95% CI) BMI by gender and Indigenous Status

Indigenous Status	Boys (n = 91)			Girls (n = 121)		
	
	BMI	n	**p-value**^**1**^	BMI	n	**p-value**^**1**^
Aboriginal and Torres Strait Islander	19.1	34		19.3	48	
	(11.3-26.9)		0.33	(12.2-26.4)		0.28
Non-Indigenous	19.9	57		20.2	73	
	(13.9-25.6)			(11.6-28.8)		

^1 ^ANOVA

### Mean Nutrient Intake

Mean nutrient intake for Aboriginal and Torres Strait Islander boys was statistically significantly higher than for non-Indigenous boys for energy, total fat, monounsaturated fat, carbohydrate, sugars, starches, fibre and sodium (Table 2). Except for fibre (*p *= 0.05), these differences were maintained by Indigenous status when adjusted for age, gender and clustering within schools. Mean intake of saturated fatty acids (*p *= 0.02), magnesium (*p *= 0.02) and potassium (*p *= 0.04) became statistically significantly higher for Aboriginal and Torres Strait Islander children compared with their non-Indigenous counterparts when Indigenous status was adjusted for age, gender and clustering within schools [see Additional File 1, **Table S1 **for all results].

**Table 2:** Mean (95% CI) daily intake of macro-nutrients, fibre, sodium and energy and differences by gender and Indigenous status^1^

Nutrient	Boys (n = 93)			Girls (n = 122)			Indigenous Status
	
	Aboriginal and Torres Strait Islander (n = 34)	Non-Indigenous (n = 59)	**p-value**^**2**^	Aboriginal and Torres Strait Islander (n = 48)	Non-Indigenous (n = 74)	**p-value**^**2**^	**p-value**^**3**^
Energy (kJ)	9689.2	8422.1	0.004	8353.5	8061.5	0.36	< .0001
	(8940.3-10438.1)	(7996.9-8847.3)		(7803.4-8903.5)	(7731.2-8391.8)		
Protein (g)	87.2	79.1	0.12	69.1	71.6	0.47	0.14
	(77.8-96.6)	(74.8-83.5)		(63.6-74.5)	(67.2-76.0)		
Total fat (g)	87.4	78.9	0.03	78	75.9	0.56	0.01
	(81.4-93.3)	(74.0-83.7)		(72.2-83.8)	(71.6-80.2)		
Saturated fatty acids (g)	38.5	34.8	0.05	34.3	33.6	0.69	0.02
	(35.7-41.4)	(32.4-37.2)		(31.3-37.3)	(31.3-35.8)		
Polyunsaturated fat (g)	10.8	10.1	0.31	10.3	9.7	0.36	0.16
	(9.8-11.7)	(9.1-11.1)		(9.2-11.3)	(9.0-10.4)		
Monounsaturated fat (g)	31.1	27.7	0.03	27.4	26.7	0.59	0.02
	(28.7-33.6)	(25.9-29.5)		(25.3-29.5)	(25.2-28.2)		
Carbohydrate (g)	289.1	241.7	0.01	251	234.9	0.15	< .0001
	(257.9-320.3)	(226.3-257.2)		(231.9-270.2)	(223.7-246.0)		
Sugars (g)	148	122.1	0.04	134	124.6	0.27	0.0002
	(125.7-170.4)	(110.4-133.8)		(119.4-148.6)	(115.4-133.7)		
Starch (g)	139.7	118.8	0.003	116.2	109.3	0.16	0.003
	(128.0-151.4)	(111.3-126.2)		(107.7-124.6)	(104.6-114.0)		
Fibre (g)	21.6	17.9	0.02	16.6	17.2	0.48	0.05
	(18.8-24.5)	(16.6-19.2)		(15.0-18.1)	(16.2-18.2)		
Sodium (mg)	2934.5	2396.9	0.01	2323.5	2341.6	0.88	0.009
	(2588.3-3280.7)	(2230.5-2563.4)		(2132.1-2514.9)	(2201.9-2481.3)		

^1 ^See Additional File 1 [Table S1] for complete table; ^2 ^Kruskall-Wallis test for difference conducted for Vitamin A, Beta-carotine, Folate, Vitamin C, Calcium and Zinc as data not normally distributed. All other nutrients examined by t-tests adjusted for clustering within schools; ^3 ^Difference by Indigenous status examined using Friedman's test for Vitamin A, Beta-carotine, Folate, Vitamin C, Calcium and Zinc as data not normally distributed and no method of adjusting for clustering. Difference by Indigenous status examined for all other nutrients by linear regression analysis adjusted for clustering within schools, age and gender.

### Percent Contributions of Macro-Nutrients to Energy

The mean percentage contribution of macronutrients to energy intake for Aboriginal and Torres Strait Islander children was generally similar to that of their non-Indigenous counterparts for both boys and girls (Table 3). Saturated fatty acid contribution to energy was approximately 15% for all children.

**Table 3:** Mean percent (95%) contribution of macronutrients to energy

Macronutrient	Boys (n = 93)			Girls (n = 122)		
	
	Aboriginal and Torres Strait Islander (n = 34) %	Non-Indigenous (n = 59) %	**p-value**^**1**^	Aboriginal and Torres Strait Islander (n = 48) %	Non-Indigenous (n = 74) %	**p-value**^**1**^
Protein	15.3	16.2	0.16	14.1	15.2	0.09
	(14.4-16.3)	(15.3-17.1)		(13.3-15.0)	(14.4-15.9)	
Fat	33.8	34.6	0.44	34.5	34.7	0.86
	(32.4-35.3)	(33.1-36.1)		(33.4-35.6)	(33.2-36.1)	
Saturated fatty acids	14.9	15.2	0.47	15.2	15.3	0.83
	(14.3-15.5)	(14.5-15.8)		(14.6-15.8)	(14.6-15.9)	
Monounsaturated fat	12.1	12.2	0.80	12.1	12.2	0.78
	(11.2-12.9)	(11.5-12.9)		(11.6-12.7)	(11.7-12.7)	
Polyunsaturated fat	4.2	4.5	0.27	4.5	4.5	0.77
	(3.9-4.5)	(4.1-4.8)		(4.0-5.1)	(4.1-4.8)	
Carbohydrate	50.2	48.6	0.13	51	49.6	0.18
	(48.6-51.8)	(47.1-51.8)		(49.2-52.8)	(48.1-51.2)	
Sugars	23.8	22.9	0.23	25.4	24.6	0.56
	(22.8-24.7)	(22.0-23.9)		(22.8-27.9)	(23.0-26.3)	
Starch	24.7	24.1	0.45	23.9	23.3	0.50
	(23.5-26.0)	(23.0-25.1)		(22.1-25.7)	(22.5-24.0)	

^1 ^Wald test from linear regression model including age and gender and adjusting for clustering of children within schools.

### Proportion of Children Meeting Australian Nutrient Reference Values for Nutrient Intake

Many children did not meet the Australian Nutrient Reference Values (NRVs) for nutrient intake for fibre, sodium, potassium and calcium (at age 12-13 years; this increased with age) (Table 4). Statistically significant differences in proportions meeting NRVs by gender were apparent for fibre (boys > girls), and phosphorus, magnesium and folate (girls > boys). No differences were found by Indigenous status alone [see Additional File 2, **Table S2 **for all results].

**Table 4:** Percent (%)^2 ^of participants with mean daily intake of dietary fibre, calcium, potassium and sodium less than Adequate Intake^3 ^or greater than the Upper Limit^4 ^(n = 215)^1^

Nutrient		Australian Nutrient Reference Values (9-13 years)		Indigenous status		
		
		Boys	Girls	Aboriginal and Torres Strait Islander (n = 82) %	Non-Indigenous (n = 133) %	**x**^**2**^ p-value
Dietary fibre^3^		24 g	20 g	77	79	0.74
Calcium	9-11 yrs^5^	800 mg		65	60	0.54
	12-13 yrs^6^	1050 mg		86	80	0.67
Potassium^3^		3000 mg	2500 mg	62	66	0.43
Sodium^4 ^		2000 mg		74	70	0.56

^1 ^See Additional File 2 [Table S2] for complete table; ^2 ^Adjusted for clustering within schools; ^3 ^Fibre and Potassium less than Adequate Intake; ^4 ^Sodium greater than the Upper Limit; ^5 ^n = 166; 6 n = 49.

### Foods Contributing to Nutrients

**Figure 2:**
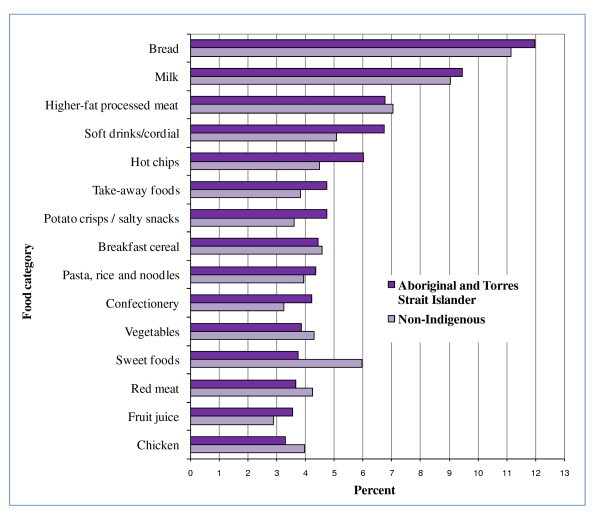
**Top 15 food categories % contribution to ENERGY intake as ranked for Aboriginal and Torres Strait Islander children**.

**Figure 3:**
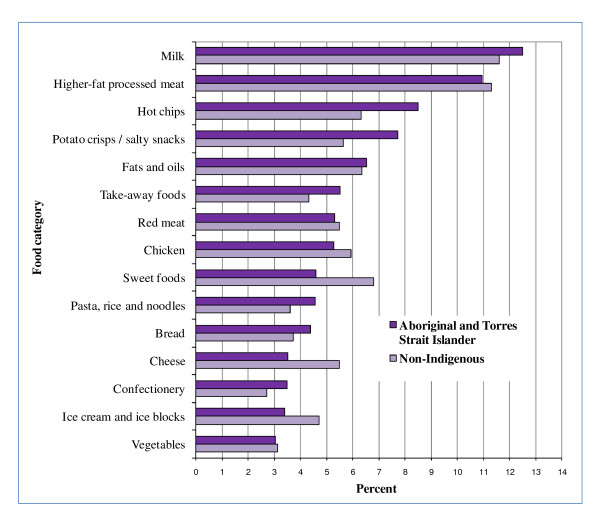
**Top 15 food categories % contribution to FAT intake as ranked for Aboriginal and Torres Strait Islander children**.

**Figure 4:**
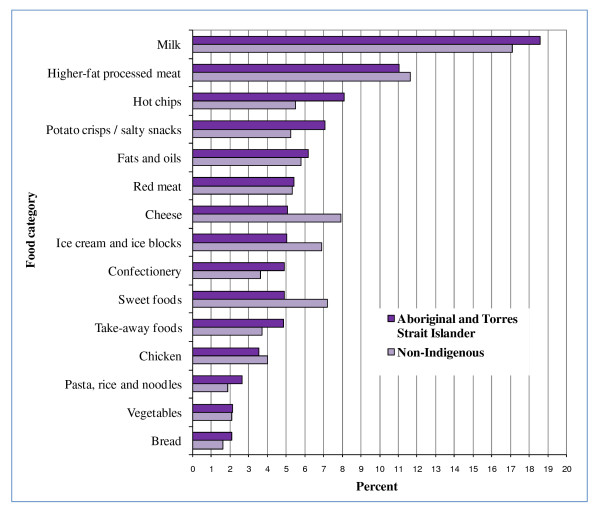
**Top 15 food categories % contribution to SATURATED FATTY ACID intake as ranked for Aboriginal and Torres Strait Islander children**.

**Figure 5:**
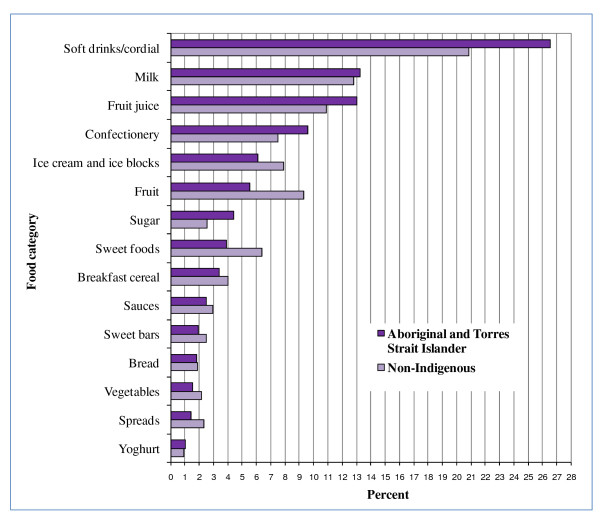
**Top 15 food categories % contribution to SUGARS intake as ranked for Aboriginal and Torres Strait Islander children**.

**Figure 6:**
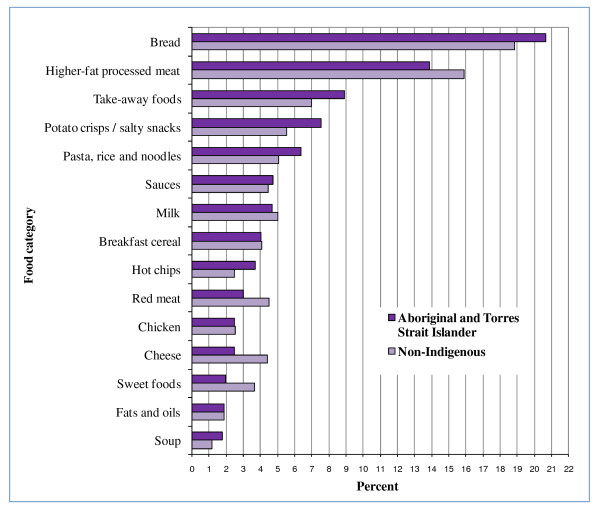
**Top 15 food categories % contribution to SODIUM intake as ranked for Aboriginal and Torres Strait Islander children**.

**Figure 7:**
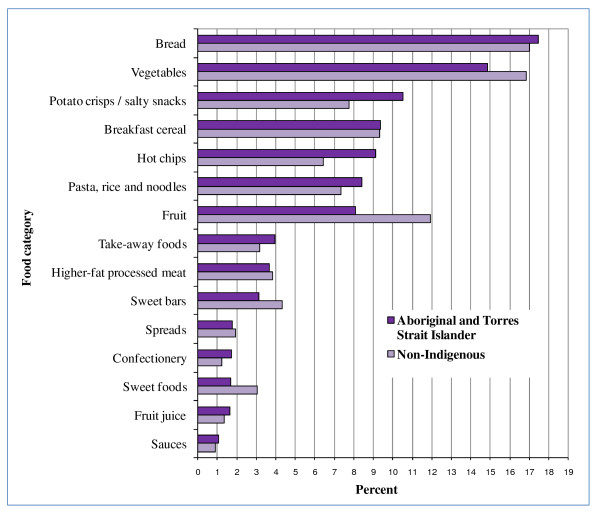
**Top 15 food categories % contribution to FIBRE intake as ranked for Aboriginal and Torres Strait Islander children**.

Many of the top 5 food categories contributing to energy, fat, saturated fat, sugar, sodium and fibre for all children were EDNP or poorer choice foods (Figures 2, 3, 4, 5, 6, and 7). Aboriginal and Torres Strait Islander girls consumed less *per capita *of vegetables and fruit, and Aboriginal and Torres Strait Islander boys in particular consumed more *per capita *of take-away meals, hot chips, potato crisps, fruit juice, and bread (Table 5). Regardless of gender Aboriginal and Torres Strait Islander children consumed notably more of soft drinks/cordial/sports drinks which were the greatest *per capita *contributors to daily food intake for all children [see Additional Files 3 and 4, **Tables S3 and S4 **for all results which also include the highest ranked food item within food category].

**Table 5:** Daily *per capita *quantity (g) and per eating occasion consumption (g) of 12 highest ranking food categories (contributing to energy, sodium, sugar, fat, and saturated fat) by Indigenous status and gender.^1^

Food category	Boys (n = 93)				Girls (n = 122)			
	
	Aboriginal and Torres Strait Islander (n = 34)		non-Indigenous (n = 59)		Aboriginal and Torres Strait Islander (n = 48)		non-Indigenous (n = 74)	
	
	*Per capita*	**E/O**^**2**^	*Per capita*	**E/O**^**2**^	*Per capita*	**E/O**^**2**^	*Per capita*	**E/O**^**2**^
Breads	**116**	85	**93**	72	**97**	68	**85**	62
Milk	**284**	218	**308**	218	**281**	218	**245**	210
Soft drinks, cordial, sports drinks	**457**	259	**319**	225	**431**	227	**279**	205
Higher-fat processed meat	**76**	95	**63**	91	**51**	86	**53**	96
Take-away meals^3^	**62**	192	**34**	161	**26**	121	**28**	145
Fruit juice	**235**	273	**142**	289	**189**	252	**163**	252
Hot chips	**58**	121	**33**	109	**38**	104	**31**	100
Sweet biscuits/cakes/muffins	**17**	65	**31**	55	**26**	57	**37**	56
Potato crisps and other salty snacks	**22**	31	**14**	26	**17**	25	**14**	23
Confectionery	**24**	33	**21**	30	**25**	26	**20**	28
Fruits	**95**	129	**97**	135	**58**	125	**114**	120
Vegetables	**131**	82	**118**	69	**96**	73	**134**	72

^1 ^See Additional Files 3 and 4 [Tables S3 and S4] for complete tables which include all highest ranking food categories and highest ranked food item within each category, by gender and Indigenous status; ^2 ^Eating occasion; ^3 ^Take-away meals such as burgers/pizza.

The highest ranked food category contributing to energy was bread (Figure 2) for all children, with white bread the highest ranking food item within this category at about 92% of consumption occasions (data not shown). EDNP foods contributed between 45% and 49% to energy for all (Table 6) [**s**ee Additional File 5, **Table S5 **for complete definition of each food category].

**Table 6:** Mean percent (%) contribution of EDNP food categories to energy by Indigenous status and gender.

		Percent contribution					
		
**Rank**^**1**^	**Food Category**^**2**^	Boys (n = 93)			Girls (n = 122)		
		
		Aboriginal and Torres Strait Islander (n = 34)	Non-Indigenous (n = 59)	**p-value**^**3**^	Aboriginal and Torres Strait Islander (n = 48)	Non-Indigenous (n = 74)	**p-value**^**3**^
1	Higher-fat processed meat	6.9	7.5	0.93	6.2	6.8	0.68
2	Hot chips	6.7	4.5	0.69	5.4	4.6	0.26
3	Soft drinks/cordial and sports drink	6.3	5.2	0.30	6.9	4.9	0.08
4	Take-away meals	6.0	4.1	0.01	3.3	3.7	0.41
5	Potato crisps and other salty snacks	5.0	3.5	0.62	4.6	3.7	0.04
7	Confectionery, lollies and chocolate	3.6	3.3	0.06	4.7	3.2	0.45
8	Sweet foods	2.6	5.1	0.31	4.8	6.7	0.79
9	Ice cream and ice blocks	2.5	2.9	0.71	3.3	4.6	0.49
10	Fats	1.9	2.3	0.26	2.6	2.2	0.26
11	Sweet bars	1.7	1.8	0.52	1.5	2.1	0.19
12	Spreads	1.6	1.8	0.69	1.6	1.7	0.47
13	Sugar	1.5	0.6	0.46	0.8	0.6	0.80
14	Sauces	0.8	1.2	0.02	1.2	1.6	0.22
15	Savoury biscuit	0.5	1.0	0.01	1.3	1.5	0.39
16	Pastry	0.0	0.5	N/A^4^	0.3	0.4	0.02

	TOTAL	47.6	45.3		48.5	48.3	

^1 ^According to that for Aboriginal and Torres Strait Islander boys; ^2 ^See Additional File 5 [Table S5] for definitions of Food Categories; ^3 ^Adjusted for clustering of children within schools; ^4 ^Not applicable.

The contribution of take-away foods and potato crisps/salty snacks was significantly higher among Aboriginal and Torres Strait Islander boys (*p *= 0.01) and girls (*p *= 0.04) respectively when compared with their non-Indigenous counterparts. Of the five highest ranking food categories contributing to fibre (Figure 7) for Aboriginal and Torres Strait Islander children, three are EDNP or poorer choice foods, and the percent contribution of fruit (8%) is lower than for non-Indigenous children (12%).

Aboriginal and Torres Strait Islander children consumed less sweet biscuits/cakes/muffins than their counterparts regardless of gender.

## Discussion

This study is one of very few internationally to comprehensively examine the food and nutrient intake of either Indigenous children or of children from disadvantaged rural regions generally. Aboriginal and Torres Strait Islander boys in this cohort demonstrate a statistically significantly higher intake of energy, most macro-nutrients and sodium when compared with non-Indigenous boys, a magnitude of difference not apparent in girls. The saturated fat mean percent contribution to energy is high for all children at about 5% above the Australian recommended range [29] and about 1.6% above the contributions found in other recent studies of children and adolescents [26,30,31], with intakes significantly higher for Aboriginal and Torres Strait Islander children than for their non-Indigenous counterparts. More than 70% of children consumed less than the EAR for fibre and calcium (at age 12-13 years) with these proportions being greater than for national data [26]. Seventy percent and higher consumed more than the UL for sodium and mean intake was similar to national data for Aboriginal and Torres Strait Islander boys (2934.5 mg) and all girls (2334.5 mg). The proportions of girls not meeting EAR for folate and magnesium, is three times that of the national population [26]. These results are consistent with international studies of children from low socio-economic regions [32-34]. There is an association between poor dietary intake of these nutrients and the development of cancer, cardiovascular disease, type 2 diabetes, other chronic diseases and neural tube defects [31,35-38], all of which occur at higher rates in Aboriginal and Torres Strait Islander communities [2,3].

Intakes of EDNP foods are excessive for all children, with contributions to daily energy intake substantially greater than Australian recommendations (14-17%) [39] for children aged 2 to 18 years [40], and than those of the only recent national study of EDNP food intake for children aged 9 to 13 years (38%) [41]. Some caution needs to be exercised when comparing results across studies as definitions of EDNP foods vary [42].

Compared with national data at 9-13 years of age [26] mean daily energy intake in this study is slightly lower for boys overall at 8885.3 kJ compared with 9645.8 kJ, however is similar for Aboriginal and Torres Strait Islander boys (9689.8 kJ). Girls mean energy intake is comparable at 8176.4 kJ overall compared with 8166.6 kJ in the national study. Difference may be due to different methods, with the data for the national study being obtained from two 24 hour recall compared with the three administered in this study.

White bread is the highest ranked food item in the top food category (bread) contributing to energy. Although bread is a 'core food'[40]. more nutrient-dense versions of this very common food item would be preferable [43] given the study population's poor dietary profile and the consequent proportions of children not meeting NRVs for many nutrients.

Higher intakes of EDNP and poorer choice foods, energy, carbohydrate, total fat and saturated fatty acids amongst Aboriginal and Torres Strait Islander boys in particular, and lower intakes of fruit and vegetables amongst Aboriginal and Torres Strait Islander girls compared with their non-Indigenous counterparts is apparent and of clinical importance. These findings are similar to international studies [44-48]. The significantly higher macro-nutrient intake of Aboriginal and Torres Strait Islander boys compared with non-Indigenous boys in our study appears to be driven by their higher consumption levels of EDNP and poorer choice foods. Aboriginal and Torres Strait Islander children who participated in this study have been shown to have a tendency to be more active than their non-Indigenous counterparts, with boys more active than girls [23]. This is likely to result in higher energy requirements and the purchase of EDNP or poorer choice foods is an affordable option to satisfy children's hunger in financially stressed communities [13,14].

Aboriginal and Torres Strait Islander children demonstrate a greater mean daily *per capita *intake compared with national data in the following food categories: soft/sports drinks/cordials (457 g [boys] and 431 g [girls] - compared with 364.7 g); potato crisps/salty snacks (22 g [boys] and 17 g [girls] compared with 12.9 g); and hot chips (58 g [boys] and 38 [girls] compared with 29.3 g) [41], with these differences not apparent among non-Indigenous children. The high contribution of the latter two foods to energy for Aboriginal and Torres Strait Islander children is about twice that for national data, [41] and for hot chips this difference is similar to findings from a Canadian study [45]. The greater daily per capita intake and percent contribution to energy of soft/sports drinks/cordials by Aboriginal and Torres Strait Islander children compared with national data (about 6.7% compared with 5.4%) [41] is probably an underestimate of difference as, unlike the national data, we were unable to include 'fruit drink' (as different from 'fruit juice') in our calculations of sugary drinks. The higher consumption levels among Aboriginal and Torres Strait Islander children (about 1 4/5 cups/day) compared with non-Indigenous children (about 1 to 1 1/5 cups/day) shown in this study are concerning. This is particularly so given the significant association between high intakes (1 to 2 cups/day) of sugary drinks and the development of metabolic syndrome and Type 2 diabetes in adults [49], both of which occur at much higher rates in Australian Aboriginal and Torres Strait Islander than non-Indigenous communities [3]. The lower consumption levels of sweet biscuits/cakes/muffins by Aboriginal and Torres Strait Islander children may be due to differences in disposable income and/or preference.

Fruit and vegetable (mostly mashed potato) intake is low for all children compared with a national survey [26] and appears lower for Aboriginal and Torres Strait Islander girls than for their non-Indigenous counterparts. Overall these findings align with those from national and state reports [50,51].

There are many factors which may impact on the poor dietary profile of children in this study including: convincing evidence that families under financial stress are unable to afford healthy foods [13,14], and that numerous environmental attributes (for example higher proportion of 'fast food' outlets in disadvantaged areas) create a more risky environment for low socio-economic families [52]. These factors have been reported by community focus groups conducted as part of our broader program of research [11].

In the context of the poorer health status and higher levels of disadvantage experienced by Aboriginal and Torres Strait Islander communities in Australia, the differences in key food and nutrient intake found in this study are important to note and warrant further investigation. A selection bias may exist in this study with relatively low and differential participation rates, the latter possibly a result of the support provided to Aboriginal and Torres Strait Islander communities by AHWs in the recruitment stage. The low participation rate of non-Indigenous children may reflect a bias towards families with better health habits, thereby leading to a falsely high difference between Aboriginal and Torres Strait Islander and non-Indigenous children. Never the less, both groups fare poorly compared with Australian NRV's, suggesting that the poor nutrient intakes documented in this study are likely understated, ie biased towards the null. The sample size limits power to detect differences when comparing proportions meeting NRVs. There is also the possibility of a differential Hawthorne effect, with Aboriginal and Torres Strait Islander children potentially over- or under-reporting due to involvement of their local communities and AHWs. However, we believe that the involvement of the Aboriginal and Torres Strait Islander community addressed a pre-existing inequity where Aboriginal and Torres Strait Islander children, due to a lack of appropriate cultural support, may have under-reported in other dietary assessments devised and supervised by the dominant culture, and been reluctant to participate in studies.

The 24-hour recall method relies on child self-report which has been shown to be as reliable as parental report by age 10 years, although there may be some difficulties in quantifying portion size at this age [53]. Recall may be influenced by the retention interval, interview format, prompts used, and correlates such as gender, BMI, and age [54]. To mitigate against this we have used the mean of 3 24-hour recalls, thus providing more robust data from this method, which may be more appropriate to use cross-culturally and with low-income participants [55] as well as with those who may be cautious about engaging in a study or for whom literacy is an issue [56]. Data were collected from children in three coastal areas and in one season during the year, and this may limit the generalisability of the findings. In addition, at the time of data entry, the latest version of the AusNut (2007) was not available, and so we used the older (1999) version, with some additions for the two key nutrients known to have changed in the food supply. However, it is possible that some other nutrients also changed in that period, and this may further limit comparisons to nutrient data provided by more recent surveys, such as the 2007 Children's Survey. However, the comparisons by foods and food groups are still possible, without concern for this potential limitation.

## Conclusions

All rural children in this disadvantaged population showed high intake levels of energy-dense and/or nutrient-poor foods and substantial proportions consumed less than EAR for important nutrients. Targeted intervention strategies need to be developed to address rural children's high consumption of white bread, processed meats, hot chips, soft drink and salty snacks, as well as these foods ready availability. Differences in intake by Indigenous status need further investigation particularly for Aboriginal and Torres Strait Islander boys, as do differences in nutrient intake and contributions of EDNP foods to key nutrients in the context of disadvantage, financial stress, and current health status experienced by Australia's Indigenous people. These findings are cause for concern for the wellbeing of Aboriginal and Torres Strait Islander children, who will continue to be at a higher risk than their non-Indigenous counterparts of developing chronic diseases such as diabetes as they become older.

## Competing interests

The authors declare that they have no competing interests. A grant of $AUD5000.00 was received from Eli Lilly, and had no role in study design, data collection/analysis, interpretation of results nor production of this manuscript.

## Authors' contributions

JG and VF contributed to conception and design of the study. JG, NT and JC collected the 24-hour recall data. JG, VF, JCYL, and CD contributed to data analysis. JG drafted the manuscript. All authors were involved in the preparation of the final manuscript.

## Pre-publication history

The pre-publication history for this paper can be accessed here:

http://www.biomedcentral.com/1471-2431/12/12/prepub

## Supplementary Material

Additional file 1 **TABLE S1: Mean daily intake of micro- and macro-nutrients, fibre and energy and differences by gender and Indigenous status**. Mean (95% CI) daily intake of micro- and macro-nutrients, fibre and energy and differences by gender and Indigenous status. Click here for file

Additional file 2 **TABLE S2: Percent of participant with mean daily intake of nutrients and fibre less than the Australian Nutrient Reference Values**. Percent (%) of participants with mean daily intake of nutrients and fibre less than Estimated Average Requirement, or less than Adequate Intake or greater than the Upper Limit (n = 215). Click here for file

Additional file 3 **TABLE S3: Daily per capita quantity and per eating occasion consumption of highest ranking food categories - boys**. Daily per capita quantity (g) and per eating occasion consumption (g) of highest ranking food categories (contributing to energy, sodium, sugar, fat, and saturated fat) and highest ranked food item within food category, for BOYS by Indigenous status. Click here for file

Additional file 4 **TABLE S4: Daily per capita quantity and per eating occasion consumption of highest ranking food categories - girls**. Daily per capita quantity (g) and per eating occasion consumption (g) of highest ranking food categories (contributing to energy, sodium, sugar, fat, and saturated fat) and highest ranked food item within food category, for GIRLS by Indigenous status. Click here for file

Additional file 5 **TABLE S5: Mean percent contribution of EDNP food categories with definitions**. Mean percent (%) contribution of EDNP food categories to ENERGY by Indigenous Status and Gender. Click here for file

## References

[B1] Hill K Barker B Vos T Excess Indigenous mortality: are Indigenous Australians more severely disadvantaged than other Indigenous populations? Int J Epidemiol 2007 36 580 589 10.1093/ije/dym011 17405802

[B2] National Health and Medical Research Council Nutrition in Aboriginal and Torres Strait Islander Peoples: An Information Paper 2000 Canberra (Australia): National Health and Medical Research Council

[B3] Australian Bureau of Statistics, Australian Institute of Health and Welfare The health and welfare of Australia's Aboriginal and Torres Strait Islander peoples 2008 2008 Canberra (Australia). Australian Bureau of Statistics

[B4] Craig ME Femia G Broyda V Lloyd M Howard NJ Type 2 diabetes in Indigneous and non-Indigenous children and adolescents in New South Wales Med J Aust 2007 186 497 499 17516894 10.5694/j.1326-5377.2007.tb01021.x

[B5] Sellars EA Moore K Dean HJ Clinical management of Type 2 Diabetes in Indigenous Youth Pediatr Clin N Am 2009 56 1441 1459 10.1016/j.pcl.2009.09.013 19962030

[B6] Mikkla V Rasanen L Raitakari OT Pietinen P Viikari J Longitudinal changes in diet from childhood into adulthood with respect to risk of cardiovascular diseases: The Cardiovascular Risk in Young Finns Study Eur J Clin Nutr 2004 58 1038 1045 10.1038/sj.ejcn.1601929 15220946

[B7] Ruben AR Undernutrition and obesity in Indigenous children: Epidemiology, prevention, and treatment Pediatr Clin N Am 2009 56 1285 1302 10.1016/j.pcl.2009.09.008 19962022

[B8] Anderson I Crengle S Leialoha Kamaka M Chen T-H Palafox N Jackson-Pulver L Indigenous health in Australia, New Zealand, and the Pacific The Lancet 2006 367 1775 1785 10.1016/S0140-6736(06)68773-4 16731273

[B9] Lee AJ Leonard D Moloney AA Minniecon DL Improving Aboriginal and Torres Strait Islander nutrition and health Med J Aust 2009 190 547 548 19450198 10.5694/j.1326-5377.2009.tb02559.x

[B10] National Aboriginal and Torres Strait Islander Nutrition Strategy and Action Plan http://www.healthinfonet.ecu.edu.au/health-risks/nutrition/policies-strategies/natsinsap

[B11] Cochrane S Community Focus Groups on Food and Physical activity availability and issues in rural NSW communities: Many Rivers Diabetes Prevention Project CRIAH Aboriginal Health Conference 2008 Sydney. Australia: Sax Institute

[B12] National Aboriginal and Torres Strait Islander Social Survey http://abs.gov.au/AUSSTATS/abs@.nsf/mf/4714.0/

[B13] Drewnowski A Obesity, diets, and social inequalities Nutr Rev 2009 67 S36 S39 19453676 10.1111/j.1753-4887.2009.00157.x

[B14] Kettings C Sinclair AJ Voevodin M A health diet consistent with Australian health recommendations is too expensive for welfare-dependent families Aust N Z J Public Health 2009 33 566 572 10.1111/j.1753-6405.2009.00454.x 20078575

[B15] Fremantle F Zurynski YA Mahajan D D'Antoine H Elliott EJ Indigenous child health:urgent need for improved data to underpin better health outcomes Med J Aust 2008 188 588 591 18484933 10.5694/j.1326-5377.2008.tb01797.x

[B16] Mackerras DE Reid A Sayers SM Singh GR Bucens IK Flynn KA Growth and morbidity in children in the Aboriginal Birth Cohort Study: the urban-remote differential Med J Aust 2003 178 56 60 12526722 10.5694/j.1326-5377.2003.tb05063.x

[B17] Trifonopouolos M Kuhnlein HV Receveur O Analysis of 24-hour recalls of 164 fourth - to sixth-grade Mohawk children in Kahnawake J Am Diet Assoc 1998 98 814 816 10.1016/S0002-8223(98)00183-7 9664926

[B18] A review of dietary intake studies in children and adolescents in Australia (draft report) http://sydney.edu.au/medicine/acaorn/streams/nutrition/publications/index.php

[B19] Supporting low SES school communities http://www.lowsesschools.nsw.edu.au/default.aspx

[B20] Australian Bureau of Statistics Census of Population and Housing: Socio-Economic Indexes for Areas (SEIFA), Australia - Data only 2006 Canberra: ABS

[B21] Cole TJ Bellizzi MC Flegal KM Dietz WH Establishing a standard definition for child overweight and obesity worldwide: International survey BMJ 2000 320 1240 1243 10.1136/bmj.320.7244.1240 10797032 PMC27365

[B22] Johnson RK Driscoll P Goran MI Comparison of multiple-pass 24-hour recall estimates of energy intake with total energy expenditure determined by the doubly labelled water method in your children J Am Diet Ass 1996 96 1140 1144 10.1016/S0002-8223(96)00293-3 8906138

[B23] Gwynn JD Hardy LL Wiggers JH Smith TH D'Este CA Turner N Cochrane J Barker DJ Attia JR The validation of a self-report measure and physical activity Australian Aboriginal and Torres Strait Islander and non-Indigenous Rural Children Aust N Z J Public Health 2010 34 S57 S65 20618297 10.1111/j.1753-6405.2010.00555.x

[B24] AusNut Australian Food and Nutrient Database 1999 1999 Canberra: Food Standards Australia New Zealand

[B25] Gwynn JD Flood VM D'Este CA Attia JR Turner N Cochrane J Wiggers JH The reliability and validity of a short food frequency questionnaire among Australian Aboriginal and Torres Strait Islander and non-Indigenous rural children Public Health Nutr 2010 doi:10.1017/S1368980010001928 10.1017/S1368980010001928 20633315

[B26] Commonwealth Scientific Industrial Research Organisation, University of South Australia 2007 Australian National Children's Nutrition and Physical Activity Survey 2008 Canberra (Australia)

[B27] Goldberg GR Black AE Jebb SA Cole TJ Murgatroyd PR Coward WA Prentice AM Critical evaluation of energy intake data using fundamental prinicples of energy physiology.1. Derivation of cut-off values to identify under-recording Eur J Clin Nutr 1991 45 569 581 1810719

[B28] Black AE Critical evaluation of energy intake using the Goldberg cut-off for energy intake:basal metabolic rate. A practical guide to its calculation, use and limitations Int J of Obes Relat Metab Disord 2000 24 1119 1130 10.1038/sj.ijo.0801376 11033980

[B29] National Health and Medical Research Council Nutrient Reference Values for Australian and New Zealand: Executive Summary 2006 Canberra (Australia): National Health and Medical Research Council

[B30] Lambert J Agostoni C Elmadfa I Hulshof K Krause E Livingstone B Socha P Pannemans D Samart, x00Ed Dietary intake and nutritional status of children and adolescents in Europe Br J Nutr 2004 92 Suppl 2 S147 211 15522158 10.1079/bjn20041160

[B31] O'Sullivan TA Ambrosini G Beilin LJ Mori TA Oddy WH Dietary intake and food sources of fatty acids in Australian adolescent Nutrition 2011 27 2 153 9 2009 10.1016/j.nut.2009.11.019 20338727

[B32] Treviño RP Fogt DL Wyatt TJ Leal-Vasquez L Sosa E Woods C Diabetes Risk, Low Fitness, and Energy Insufficiency Levels among Children from Poor Families J Am Diet Assoc 2008 108 1846 1853 10.1016/j.jada.2008.08.009 18954574

[B33] Langevin DD Kwiatkowski C McKay MG Maillet JOS Touger-Decker R Smith JK Perlman A Evaluation of Diet Quality and Weight Status of Children from a Low Socioeconomic Urban Environment Supports "At Risk" Classification J Am Diet Assoc 2007 107 1973 1977 10.1016/j.jada.2007.08.008 17964318

[B34] Devaney B Kim M Carriquiry A Camano-Garcia G Assessing the nutrient intakes of vulnerable subgroups: final report submitted to the USDA, Economic Research Services 2005 Princeton, N.J.

[B35] Larsson SC Wolk A Magnesium intake and risk of type 2 Diabetes: a meta-analysis J Intern Med 2007 262 208 214 10.1111/j.1365-2796.2007.01840.x 17645588

[B36] Couzos S Murray R Aboriginal primary health care: an evidence-based approach 2008 3 Melbourne, Australia: Oxford University Press

[B37] Longstreet D Heath DL Panaretto KS Vink R Correlations suggest low magnesium may lead to higher rates of type 2 diabetes in Indigenous Australians Rural Remote Health 2007 7 17937505

[B38] Sauer J Mason JB Choi SW Sauer J Mason JB Choi S-W Too much folate: a risk factor for cancer and cardiovascular disease? Curr Opin Clin Nutr Metab Care 2009 12 30 36 10.1097/MCO.0b013e32831cec62 19057184 PMC2790187

[B39] Bell AC Kremer PJ Magarey AM Swinburn BA Contribution of 'noncore' foods and beverages to the energy intake and weight status of Australian children Eur J Clin Nutr 2005 59 639 645 10.1038/sj.ejcn.1602091 15714218

[B40] Smith A Kellett E Schmerlaib Y The Australian Guide to Healthy Eating: background information for nutrition educators 1998 Canberra (Australia): Commonwealth Department of Health and Aging

[B41] Rangan AM Kwan J Flood VM Louie JCY Gill TP Changes in 'extra' food intake among Australian children between 1995 and 2007 Obes Res Clin Prac 2011 5 1 e55 63 doi:10.1016/j.orcp.2010.12.001 10.1016/j.orcp.2010.12.001 24331011

[B42] Rangan AM Randall D Hector DJ Gill TP Webb KL Consumption of 'extra' foods by Australian children: types. quantities and contribution to energy and nutrient intakes Eur J Clin Nutr 2008 62 256 364 10.1038/sj.ejcn.1602720 17356553

[B43] Dietary Guidelines for Children and Adolescents in Australia http://www.nhmrc.gov.au/_files_nhmrc/publications/attachments/n34.pdf

[B44] Sluyter JD Schaaf D Metcalf PA Scragg RK Dietary intakes of Pacific, Maori, Asian and European adolescents: the Auckland High School Heart Survey Aust N Z J Public Health 2010 34 10.1111/j.1753-6405.2010.00470.x 20920102

[B45] Taylor Jp Timmons V Larsen R Walton F Bryanton J Critchley K McCarthy MJ Nutritional concerns in Aboriginal children are similar to those in non-Aboriginal children in Prince Edward Island J Am Diet Assoc 2007 107 951 955 10.1016/j.jada.2007.03.008 17524715

[B46] Garriguet D Obesity and the eating habits of the Aboriginal population Health Rep 2008 19 21 35 18457209

[B47] Receveur O Morou K Gray-Donald K Macaulay AC Consumption of Key Food Items Is Associated with Excess Weight among Elementary-School-Aged Children in a Canadian First Nations Community J Am Diet Assoc 2008 108 362 366 10.1016/j.jada.2007.09.002 18237583

[B48] Stroehla BC Malcoe LH Velie EM Dietary Sources of Nutrients among Rural Native American and White Children J Am Diet Assoc 2005 105 1908 1916 10.1016/j.jada.2005.09.002 16321596

[B49] Malik VS Popkin BM Bray GA Despres J-P Willett WC Hu FB Sugar-sweetened beverages and risk of metabolic syndrome and type 2 diabetes Diabetes Care 2010 33 2477 2483 10.2337/dc10-1079 20693348 PMC2963518

[B50] Zubrick S Lawrence D Silburn S Blair E Milroy H Wilkes T Eades S D'Antoine h Read A Ishiguchi P Doyle S The Western Australian Aboriginal Child Health Survey: The Health of Aboriginal Children and Young People 2004 Perth (Australia): Telethon Institute for Child Health Research

[B51] National Aboriginal and Torres Strait Islander health survey http://www.abs.gov.au/ausstats/abs@.nsf/mf/4715.0

[B52] Yancey AK Kumanyika SK Bridging the Gap: Understanding the Structure of Social Inequities in Childhood Obesity Am J Prev Med 2007 33 S172 S174 10.1016/j.amepre.2007.07.013 17884564

[B53] Livingstone MB Robosn PJ Wallace JM Issues in dietary intake assessment of children and adolescents Br J Nutr 2004 92 S213 S222 10.1079/BJN20041169 15522159

[B54] Baxter SD Cognitive processes in children's dietary recalls: insight from methodological studies Eur J Clin Nutr 2009 63 S19 S32 19190640 10.1038/ejcn.2008.61 PMC2714261

[B55] Rockett HRH Berkey CS Colditz GA Evaluation of dietary assessment instruments in adolescents Curr Opin Clin Nutr Metab Care 2003 6 557 562 10.1097/00075197-200309000-00009 12913673

[B56] Cade J Burley V Warm D Thompson R Margetts B Food-frequency questionnaires: a review of their design, validation and utilisation Nutr Res Rev 2004 17 5 22 10.1079/NRR200370 19079912

